# Prognostic Implications of MicoRNA miR-195 Expression in Human Tongue Squamous Cell Carcinoma

**DOI:** 10.1371/journal.pone.0056634

**Published:** 2013-02-22

**Authors:** Ling-fei Jia, Su-bi Wei, Kai Gong, Ye-hua Gan, Guang-yan Yu

**Affiliations:** 1 Department of Oral and Maxillofacial Surgery, Peking University School and Hospital of Stomatology, Beijing, People’s Republic of China; 2 Medical Systems Biology Research Center, Tsinghua University, Beijing, People’s Republic of China; 3 State Key Laboratory of Biomembrane and Membrane Biotechnology, School of Life Sciences, Tsinghua University, Beijing, People’s Republic of China; 4 Central Laboratory, Peking University School and Hospital of Stomatology, Beijing, People’s Republic of China; Columbia University Medical Center, United States of America

## Abstract

**Background:**

miR-195 is aberrantly expressed in multiple types of disease. But little is known about the dysregulation of miR-195 in tongue squamous cell carcinoma (TSCC). In this study, we investigated the roles of miR-195 in the development and progression of TSCC.

**Methods:**

Using quantitative reverse transcription-polymerase chain reaction (qRT-PCR), we evaluated miR-195 expression in TSCC samples from 81 patients. Overall survival of these patients was examined using Kaplan–Meier curves with log-rank tests and the Cox proportional hazards model. The expression of two known miR-195 target genes, Cyclin D1 and Bcl-2, was also examined in the TSCC samples by immunohistochemistry. The effects of miR-195 overexpression on cell cycle progression and apoptosis and its effects on the expression of Cyclin D1 and Bcl-2 were examined in transfected TSCC cell lines (SCC-15 and Cal27) using fluorescence-activated cell sorting assays, luciferase reporter assays, and Western blots.

**Results:**

Reduced miR-195 expression was associated with tumor size and the clinical stage of TSCC tumors. Kaplan–Meier survival analysis indicated that the TSCC patients with reduced expression of miR-195 had poor overall survival and in multivariable analyses low levels of miR-195 emerged as an independent prognostic factor for this clinical outcome. Levels of miR-195 expression were inversely correlated with the expression of Cyclin D1 and Bcl-2. Overexpression of miR-195 inhibited cell cycle progression, promoted apoptosis, and reduced Cyclin D1 and Bcl-2 expression in two TSCC cell lines.

**Conclusions:**

miR-195 may have potential applications as a prognostic factor for TSCC patients.

## Introduction

Head and neck squamous cell carcinoma, including cancers of oral cavity, oropharynx, larynx, and hypopharynx, represents the sixth most frequent solid cancer around the world [1]. Tongue squamous cell carcinoma (TSCC) is the most common type of oral cancer and is well-known for its high rate of proliferation and nodal metastasis [2]. Although TSCC is visibly located in the oral cavity, in previous studies up to 50% of patients were already in advanced stage III and IV on presentation [3], [4]. Understanding the molecular pathways of TSCC carcinogenesis and progression would be helpful in improving diagnosis, therapy, and prevention of this disease.

MicroRNAs (miRNAs) are endogenously expressed small non-coding RNAs that inhibit gene expression through the 3′-untranslated regions (3′-UTRs) of their target messenger RNAs [5]. Because of their widespread control of gene expression, miRNAs play crucial roles in numerous biological processes, including cell growth, apoptosis, metabolism, and transformation [6], [7], [8]. In TSCC, miR-184 is overexpressed and acts as an “oncogene” [9], miR-138 plays an important role in cell migration and invasion [10] and miR-21 indicates poor prognosis in TSCC patients [11]. miR-195 was first predicted based on homology to a verified miRNA from the mouse [12] and was later shown to exist in humans [13]. Recent studies have demonstrated that miR-195 expression is decreased, relative to nonmalignant tissue, in many solid tumors, including bladder cancer [14], gastric cancer [15], colorectal cancer [16], and hepatocellular carcinoma [17]. However, miR-195 expression has been reported to be increased in adrenocortical adenomas [18] and breast cancer [19]. Therefore, miR-195 may display either pro-proliferative or pro-apoptotic roles under specific physiological conditions and in different types of cancers. So far, the expression and role of miR-195 in TSCC remains to be examined.

Cyclin D1 is one of the key proteins involved in cell cycle control and is essential for G1 to S transition [20]. Bcl-2 is one of the key regulators of apoptosis and confers a survival advantage to cells by protecting them from apoptotic death [21]. Previous studies have shown that miR-195 prevents cell proliferation and promotes apoptosis in diverse cancers by binding to the 3′-UTRs of mRNAs of Bcl-2 and Cyclin D1 [16], [17]. However, the relationship between the expression of miR-195 and its target gene Cyclin D1 and Bcl-2 has not been reported in TSCC.

In this study, we found that the expression of miR-195 was statistically significantly decreased in primary TSCC compared with matched normal tissues and was associated with progression and prognosis of TSCC patients. Further analysis showed that Cyclin D1 and Bcl-2 expression were both inversely correlated with miR-195 expression and that overexpression of miR-195 inhibits cell cycle progression and promotes apoptosis of TSCC cells, probably by reducing the expression of Cyclin D1 and Bcl-2. These results suggest important roles for miR-195 in TSCC pathogenesis and implicate its potential application in cancer prognosis.

## Materials and Methods

### Ethics Statement

These experiments were approved by the Institutional Ethics Committee of Peking University School of Stomatology and all samples were obtained from patients who signed informed consent forms approving the use of their tissues for research purposes after surgery. (Approval number PKUSSIRB-2012010).

### Tissue Specimens

Paired primary TSCC samples from anterior portions of the tongue and adjacent histological normal tissues were obtained from 81 patients who were admitted to the Department of Oral and Maxillofacial Surgery of Peking University Hospital of Stomatology between May 2008 and August 2011. The median duration of follow-up was 24 months (range, 9–48 months). None of the patients received treatment before surgery. Tumor tissues and adjacent normal tissues that were at least 1.5 cm distal to the tumor margins were snap-frozen in liquid nitrogen and then stored at −80°C until use. The clinicopathological characteristics of patients are summarized in Table 1. The clinical tumor node metastasis (TNM) staging of the tumors was classified according to the standards provided by AJCC in 2010 [22]. Among 42 patients who had pathologically metastatic cervical lymph nodes, at least 9 patients were c**N**0 (clinical negative lymph nodes).

**Table 1:** Relationship between expression of miR-195, Cyclin D1, and Bcl-2 and clinicopathologic factors in 81 TSCC patients.

		miR-195 (T/N)		Cyclin D1(% )			Bcl-2 (% )		
Characteristics	No.	Mean ± SD	*P*	No. of low expression	No. of high expression	*P*	No. of low expression	No. of high expression	*P*
Sex			0.739			0.397			0.250
Male	45	0.625±0.623		22	23		27	18	
Female	36	0.674±0.693		21	15		26	10	
Age			0.321			0.397			0.105
<60 y	45	0.708±0.762		22	23		26	19	
≥60 y	36	0.570±0.502		21	15		27	9	
Tumor size			0.005			0.023			0.254
T_1_–T_2_	53	0.772±0.762		33	20		37	16	
T_3_–T_4_	28	0.425±0.297		10	18		16	12	
Differentiation			0.747			0.932			0.608
Well	35	0.709±0.753		18	17		23	12	
Moderate	38	0.592±0.590		21	17		26	12	
Poor	8	0.687±0.582		4	4		4	4	
Clinical stage			0.019			0.491			0.778
I–II	48	0.780±0.770		27	21		32	16	
III–IV	33	0.466±0.394		16	17		21	12	
Node metastasis			0.493			0.056			0.488
Yes	42	0.603±0.592		18	24		26	16	
No	39	0.705±0.728		25	14		27	12	
Status			0.010			0.254			0.088
Survival	48	0.810±0.755		28	20		35	13	
Death	33	0.433±0.418		15	18		18	15	

Abbreviations: T, tumor; N, nonmalignant tissue; T1–T4: T stage of TNM classification system.

### RNA Isolation and Quantitative Reverse-transcription PCR (qRT-PCR)

Total RNA, including miRNA, was isolated from tumor and normal tissue samples by using TRIzol reagent (Invitrogen, Carlsbad, CA) according to the manufacturer’s instructions. For miR-195 analysis, the stem-loop RT primer was 5′-GTC GTA TCC AGT GCA GGG TCC GAG GTA TTC GCA CTG GAT ACG ACG CCA AT-3′ and the amplifying primers were as follows: sense, 5′-CGT AGC AGC ACA GAA AT-3′ and antisense, 5′-GTG CAG GGT CCG AGG T-3′ [16]. Primers for qRT-PCR of U6, an mRNA that was used as an internal control, were:sense, 5′-CTC GCT TCG GCA GCA CA-3′ and antisense, 5′-AAC GCT TCA CGA ATT TGC GT-3′ [16]. Quantitative PCR was conducted at 95°C for 10 min followed by 40 cycles of 95°C for 15 sec and 60°C for 60 sec in an ABI 7500 real-time PCR system. The relative expression level of miR-195 was normalized to that of U6 by the 2^−ΔΔCt^ cycle threshold method [23].

### Immunohistochemistry and in situ Hybridization

Immunohistochemical staining was performed with a two-step detection kit (Zhongshan Goldenbridge, Beijing, China) as described previously [24]. The primary antibodies were Cyclin D1 (Santa Cruz, CA, 1∶100 dilution) and Bcl-2 (Invitrogen, Carlsbad, CA, 1∶200 dilution). A four-grade scoring system was used to evaluate the degree of Cyclin D1 immunostaining: score 0, <5%; score 1, 5% to 25%; score 2, 25% to 50%; score 3, >50% of tumor cells with positive immunostaining. The intensity of Bcl-2 immunoreactions was scored as follows: score 0, negative; score 1, weak; score 2 moderate; score 3, strong. Scores 0 and 1 of the immunostaining were defined as low expression, whereas scores 2 and 3 were defined as high expression. miRNAs in situ hybridization assay were performed essentially as previously described [25]. Dual-DIG-labelled LNA probes miR-195 detection probe or Scramble-miR were obtained from Exiqon (Exiqon, Vedbaek, Denmark) and the hybridizations were performed at 42°C.

### Cell Cultures and Transfection

Human tongue cancer cell lines SCC-15 and CAL27 were purchased from American Type Culture Collection (ATCC, Manassas, VA, USA), cultured at 37°C in a humidified atmosphere of 5% CO2, and fed Dulbecco’s modified Eagle’s medium (DMEM) supplemented with 10% fetal calf serum with 100 U/ml penicillin and 100 µg/ml streptomycin. The empty vector, pcDNA3.0, and an miR-195 expression vector, pcDNA3.0-miR-195, were gifts from Dr. Shi-Mei Zhuang (Sun Yatsen University, China) [17]. SCC-15 and CAL27 cells were seeded onto 6-well plates the day before transfection to ensure 80% conuence at the time of transfection. Transfection with 4 µg of pcDNA3.0 or pcDNA3.0-miR-195 and 100 nm Cyclin D1 and Bcl-2 siRNA or siRNA control were performed using Lipofectamine 2000 (Invitrogen, Carlsbad, CA) in accordance with the manufacturer’s procedure.

### Cell Proliferation Assays

The effects of miR-195 overexpression on SCC-15 and CAL27 cell proliferation were assessed using the Cell Counting Kit-8 (CCK-8, Dojindo, Kumamoto, Japan). Briey, the cells were seeded into 96-well plates (2×10^3^ cells/well). After transfection with pcDNA3.0 or pcDNA3.0-miR-195, CCK-8 (10 µl) was added to each well at various time points and incubated at 37°C for 3 h. The absorbance at 450 nm was measured using a microplate spectrophotometer (Bio-Tek Instruments Inc, Winosski, VT).

### Cell Cycle and Apoptosis Analysis

At 48 h post-transfection, cells were harvested by trypsinization and washed with phosphate-buffered saline (PBS). For cell cycle analysis, the cells were fixed with 70% ethanol at 4°C overnight. On the following day, fixed cells were washed with PBS, treated with RNase A (50 µg/ml) in PBS at 37°C for 20 min, and then mixed with propidium iodide (PI, 50 µg/ml) for 30 min in the dark. The stained cells were analyzed with fluorescence-activated cell sorting (FACS) by flow cytometry (FACSCalibur, Becton Dickinson,Bedford, MA). The cell debris and fixation artifacts were gated out and cell populations that were at the G0/G1, S, and G2/M phases were quantified using the ModFit software (Becton Dickinson). At least 10,000 cells in each sample were analyzed to obtain a measurable signal. For apoptosis analysis, an Annexin-V-FLUOS Staining kit (Roche, Mannheim, Germany) was used according to the manufacturer’s instructions 48 h after transfection. Apoptosis was analyzed with FACS using the CellQuest software (Becton Dickinson). Annexin-V-FLUOS-positive cells were regarded as apoptotic cells.

### Vector Construction and Luciferase Reporter Assay

The human Bcl-2 and Cyclin D1 3′-UTRs, which contained predicted targets of miR-195, were amplified by PCR and cloned into a modified version of pcDNA3.1(+) that contained a firefly luciferase reporter gene (gift from Brigid L.M. Hogan, Duke University, Durham, NC, USA) [26], at a position downstream of the luciferase reporter, between the *Eco*RI and *Xho*I cloning sites. The vectors were named wild type 3′UTRs and the primers for cloning the 3′-UTRs of Cyclin D1 and Bcl-2 were as follows: Cyclin D1, sense, 5′-GAT GAA TTC TTA TCC CCT GCC CCT TCC-3′ and antisense, 5′-TAT CTC GAG TGG GTC CAC CAT GGC TAA GTG A-3′; Bcl-2, sense, 5′-GAC GAA TTC AAT GCA GTG GTG CTT AC-3′ and antisense, 5′-CTT CTC GAG GAG GAG GTT CTC AGA TGT T-3′. Site-directed mutagenesis of the miR-195 binding sites in Cyclin D1 and Bcl-2 3′UTRs were performed using Site-Directed Mutagenesis Kit (SBS Genetech, Beijing, China) and named as mutant 3′UTRs. The primers for cloning the mutant 3′-UTRs of Cyclin D1 and Bcl-2 were as follows: Cyclin D1 binding site 1, sense, 5′- TTT CTT ATT GCG CAC GTA CCG TTG ACT TCC AG-3′ and antisense, 5′- CTG GAA GTC AAC GGT ACG TGC GCA ATA AGA AA -3′; Cyclin D1 binding site 2,sense, 5′- CTT TCA CAT TGT TTG GAC CTA TTG GAG GAT CAG -3′ and antisense, 5′- CTG ATC CTC CAA TAG GTC CAA ACA ATG TGA AAG -3′;Bcl-2, sense, 5′- GGA ATA TCC AAT CCT GTC GAC CTA TCC TGC CAA-3′ and antisense, 5′- TTG GCA GGA TAG GTC GAC AGG ATT GGA TAT TCC-3′. All constructs were confirmed by DNA sequencing. SCC-15 and CAL27 cells grown in a 48-well plate were co-transfected with 400 ng of either pcDNA3.0 or pcDNA3.0-miR-195, 40 ng of the firefly luciferase reporter plasmid including the 3′-UTR of the target gene, and 4 ng of pRL-TK, a plasmid expressing rellina luciferase (Promega, Madison, WI)). After 24 h, the dual-luciferase reporter assay was performed as reported [27].

### Western Blot Analysis

Cells were lysed in RIPA lysis buffer (50 mM Tris/HCl, pH 8.0, 250 mM NaCl, 1% NP40, 0.5% (w/v) sodium deoxycholate, 0.1% sodium dodecylsulfate). Lysates were sonicated and centrifuged at 12,000 g/min at 4°C for 10 min. Aliquots (50 µg) of the protein extracts were subjected to 12% SDS polyacrylamide gel electrophoresis and transferred to polyvinylidene difluoride membrane (Millipore, Billerica, MA). Membranes were incubated with primary antibodies at 4°C overnight and washed extensively, followed by incubation with horseradish peroxidase-conjugated second antibodies (Zhongshan Goldenbridge, Beijing, China, 1∶10,000 dilution) at room temperature for 1 h and detected with ECL kit (Applygen, Beijing, China). The primary antibodies, Cyclin D1 (Santa Cruz Biotechnology, Santa Cruz, CA), Bcl-2 (Cell Signaling Technology, Beverly, MA), and β-actin (Santa Cruz) were diluted 1∶1,000 respectively.

### RNA Oligoribonucleotide

The small interfering RNA (siRNA) targeting human Cyclin D1 and Bcl-2 transcripts and siRNA control were purchased from Integrated Biotech Solutions Company (Ibsbio, Shanghai, China). Sequence of these siRNAs were: Cyclin D1 siRNA, sense, 5′-CAA GCU CAA GUG GAA CCU GTT-3′, antisense, 5′-CAG GUU CCA CUU GAG CUU GTT-3′; Bcl-2 siRNA, sense, 5′-GUG AAG UCA ACA UGC CUG CTT-3′, antisense, 5′-GCA GGC AUG UUG ACU UCA CTT-3′; siRNA control, sense, 5′-UUC UCC GAA CGU GUC ACG UTT-3′, antisense, 5′-ACG UGA CAC GUU CGG AGA ATT-3′.

### Statistical Analysis

Student's *t* test and one-way ANOVA were used to analyze the relationship between miR-195 expression and clinicopathologic characteristics. The relationships between Cyclin D1 or Bcl-2 expression and clinicopathologic parameters were explored using the Pearson χ2 test. Correlation between miR-195 expression and Cyclin D1 or Bcl-2 protein levels was analyzed using Spearman’s rank correlation coefficient analysis with r and *P* values as indicated. Survival curves were constructed by the Kaplan-Meier method and the curves were compared using the log-rank test. The Cox regression model was applied to simultaneously adjust all potential prognostic variables. All statistical analyses were performed using SPSS for Windows version 16.0 (SPSS). Experiments with cell cultures were done at least in triplicate. Data were expressed as mean ± standard deviation (SD). A two-tailed value of *P*<0.05 was considered to be statistically significant.

## Results

### miR-195 Expression was Reduced in TSCC and was Correlated with Cancer Progression

**Figure 1:**
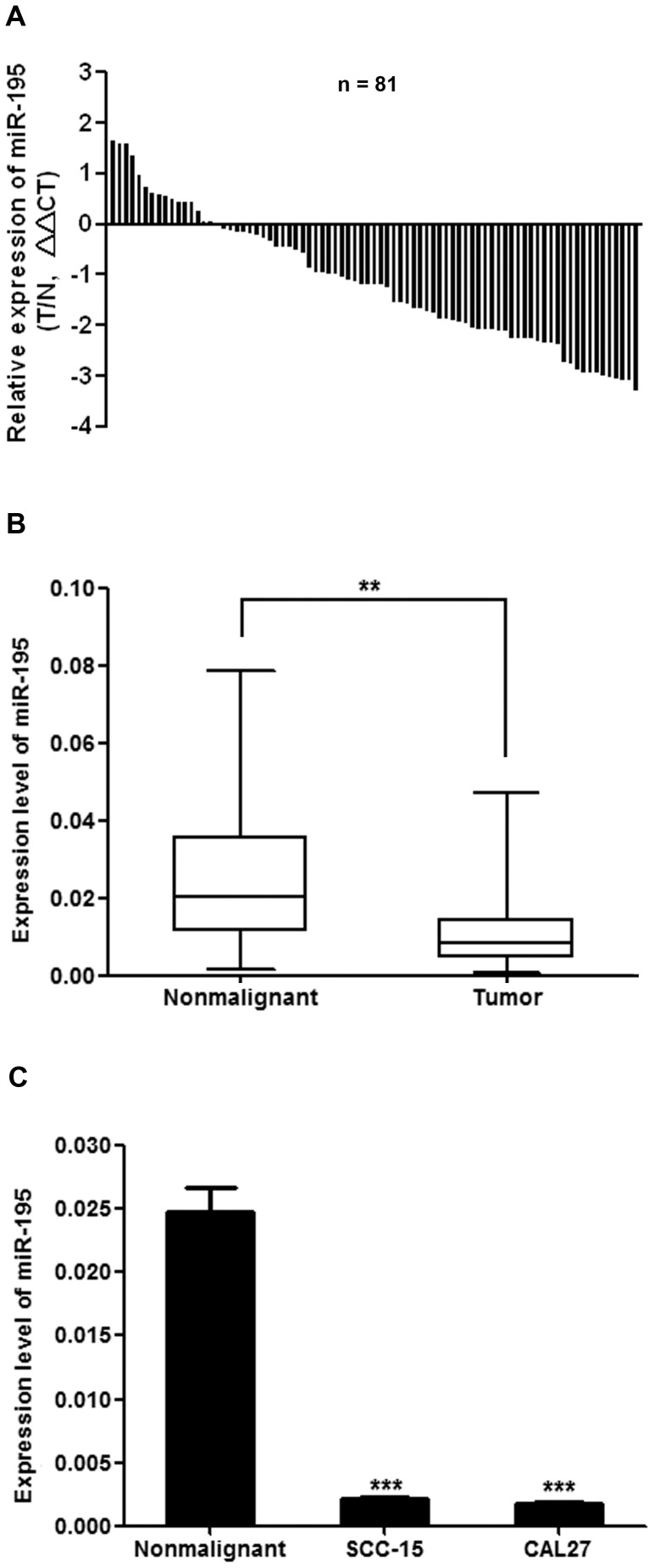
miR-195 expression was reduced in human TSCC and cell lines. (A), Relative levels of miR-195 in 81 surgical specimens of TSCC and matched adjacent nonmalignant tissues was quantified by qRT-PCR. Data were presented as log2 fold change (ΔΔCT values, TSCC/Nonmalignant, T/N). (B), Means of miR-195 relative levels for 81 surgical specimens of TSCC and the matched adjacent nonmalignant tissues. Data were presented as 2^−ΔCt (miR-195-U6)^ values (**P<0.01). (C), miR-195 expression was examined by qRT–PCR for 81 surgical specimens of nonmalignant tissues and cell lines as indicated. Data were presented as 2^−ΔCt (miR-195-U6)^ values (***P<0.001).

We evaluated the expression levels of miR-195 in 81 pairs of TSCC and the adjacent histologically normal tissues. miR-195 expression was decreased in 65 of 81 (80.2%) tumor samples compared with their nonmalignant counterparts (Fig. 1A). The average expression level of miR-195 was statistically significantly decreased in tumor tissues compared with matched nonmalignant tissues (Fig. 1B; *P*<0.01). Moreover, miR-195 expression was also statistically significantly decreased in the TSCC cell lines SCC-15 and CAL27, compared with TSCC adjacent nonmalignant tissues (Fig. 1C). To further determine whether there was an association between miR-195 expression and tumor prognosis, we looked for a correlation between miR-195 expression and the clinicopathological features of TSCC. By normalizing miR-195 expression levels in the tumor tissues with those in adjacent nonmalignant tissues (Tumor/Nonmalignant, T/N), we found that there were statistically significant relationships between miR-195 expression (T/N) and some clinicopathologic features of TSCC (Table 1), including tumor size (*P* = 0.005), clinical stage (*P* = 0.019), and patient mortality (*P = *0.010).

### Decreased miR-195 Expression was Associated with Poor Overall Survival in TSCC Patients

**Figure 2:**
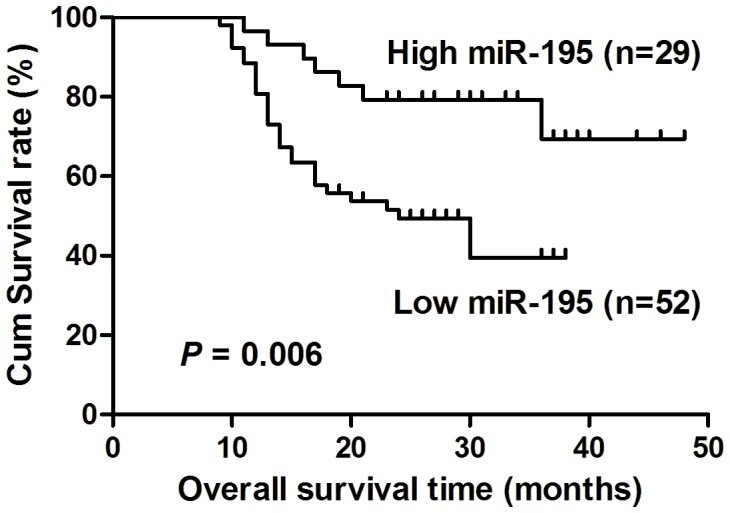
Decreased expression of miR-195 was correlated with poor survival in TSCC patients. Kaplan-Meier curves with log rank tests show that patients with high miR-195 expression (T/N fold change >0.652) survived statistically significantly longer (*P* = 0.006) than those with low miR-195 expression (T/N fold change <0.652). The median miR-195 expression level (T/N = 0.652) in the tumor samples was chosen as the cut-off point.

We further analyzed the correlation of miR-195 expression and postoperative overall survival of TSCC patients. With the mean fold change (T/N = 0.652) in miR-195 expression chosen as the cut-off point, we divided the patients into a high miR-195 expression group (T/N fold change >0.652) and a low miR-195 expression group (T/N fold change <0.652). Patients with high miR-195 expression survived statistically significantly longer than those with low miR-195 expression (Fig. 2; *P = *0.006). To identify whether miR-195 is an independent prognostic covariate for TSCC, we did a multivariable Cox proportional hazards analysis. In the final multivariable Cox regression model, low levels of miR-195 expression in TSCC were associated with a poor prognosis in terms of overall survival (*P = *0.025, relative risk = 0.322), independent of other clinical covariates (Table 2), suggesting that miR-195 might be used as an independent prognostic factor for TSCC.

**Table 2:** Multivariable analysis of various prognostic variables in TSCC patients using Cox regression analysis.

Variables	Case No.	*P*	Regression coefficient	Relative risk	95% confidence interval
Differentiation					
Well	35	0.718	0.386	1.149	0.539–2.450
Mediate & Poor	46				
Clinical stage					
I–II	48	0.194	0.376	1.630	0.780–3.408
III–IV	33				
Node metastasis					
Yes	42	0.169	0.388	1.705	0.797–3.649
No	39				
miR-195	81	0.025	0.505	0.322	0.120–0.865

### Expression of the Cyclin D1 and Bcl-2 Proteins were Both Inversely Correlated with miR-195 Expression in TSCC

**Figure 3:**
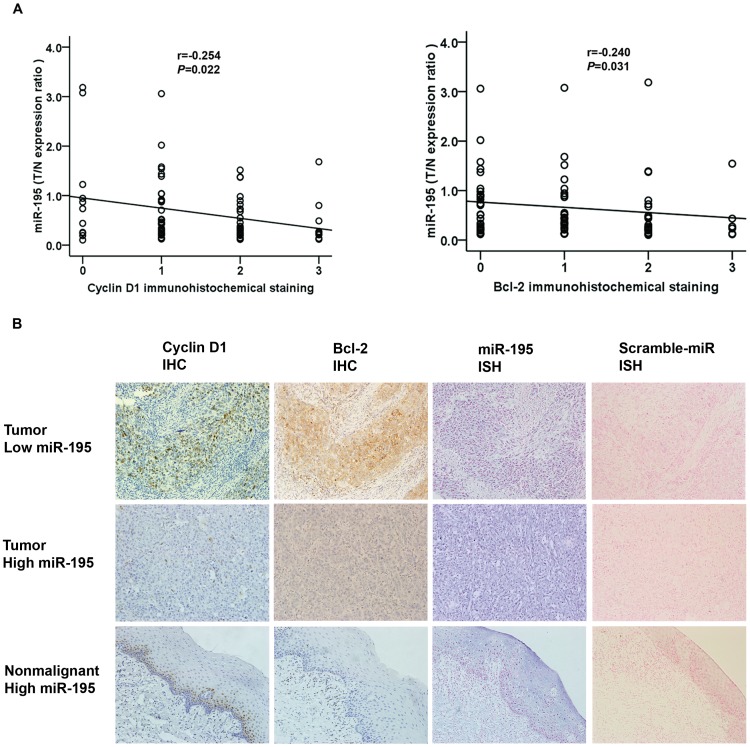
Inverse correlation between miR-195 and Cyclin D1 or Bcl-2 protein levels in TSCC. Expression of Cyclin D1 and Bcl-2 was examined by immunohistochemistry (IHC) and miR-195 expression was detected by qRT–PCR and in situ hybridization (ISH). (A), Statistical analysis of the expression of miR-195 in tumor vs nonmalignant tissue. Spearman’s rank correlation analysis was performed, with r and P values as indicated. (B), The concurrence of miR-195 expression and corresponding variation of Cyclin D1 and Bcl-2 was confirmed in human TSCC and nonmalignant specimens by ISH with miR-195 detection probe or Scramble-miR and IHC (200×magnification).

Because the Cyclin D1 and Bcl-2 transcripts were shown to be direct targets of miR-195 and their inhibition may account for the antitumor effect of miR-195 [16], [17], we examined the expression of the proteins they encode in paraffin sections of TSCC and nonmalignant samples using immunohistochemistry and Spearman’s rank correlation coefficient analysis. Levels of staining of Cyclin D1 and Bcl-2 in TSCC cancer tissues were inversely correlated with miR-195 levels (Fig. 3A). In confirmation, we examined the expression of miR-195 in paraffin sections of TSCC and nonmalignant samples using in situ hybridization. Both immunohistochemistry and in situ hybridization analysis in consecutive pathological dissections showed miR-195 expression were inversely correlated with Cyclin D1 and Bcl-2 in all three specimens examined. The Bcl-2 staining in TSCC adjacent nonmalignant tissues was generally of reduced intensity and Cyclin D1 staining was only found in basal cells of normal epithelium, coincident with the relatively high miR-195 signal (Fig. 3B). To gain an insight into the roles of Cyclin D1 and Bcl-2 in human TSCC development, we analyzed the association of the expression of Cyclin D1 and Bcl-2 with clinicopathological parameters. As shown in Table 1, the expression of Cyclin D1 was associated with tumor size of TSCC (*P* = 0.023), whereas the expression of Bcl-2 was not statistically significantly associated with any of the clinicopathologic parameters.

### Overexpression of miR-195 Inhibited Cell Cycle Progression and Promoted Apoptosis in TSCC Cell Lines

**Figure 4:**
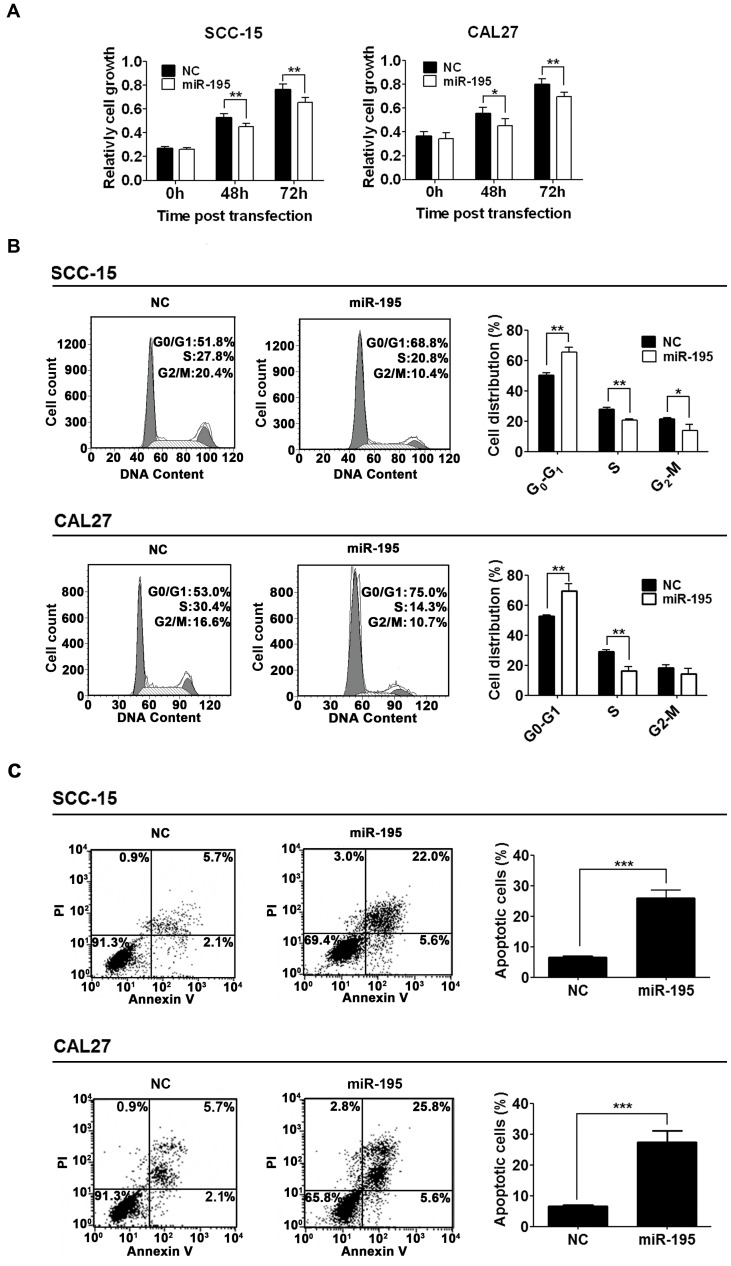
Overexpression of miR-195 inhibited cell viability and cell cycle progression and promoted cell apoptosis. (A), Inhibition of cell viability by overexpression of miR-195. SCC-15 and CAL27 cells were transfected with pcDNA3.0, a negative control (NC) or with pcDNA3.0-miR-195 (miR-195), as indicated. Cell viability was measured using CCK-8 assays. The data were presented as means ± SD (n = 5) (* *P*<0.05, ** *P*<0.01). (B), Inhibition of cell cycle progression by overexpression of miR-195. SCC-15 and CAL27 cells were transfected as in (A). Cells were stained with propidium iodide (PI) at 48 h post-transfection and analyzed with FACS (* *P*<0.05, ** *P*<0.01). (C), Promotion of apoptosis by overexpression of miR-195. SCC-15 or CAL27 cells were transfected for 48 h as in (A) and apoptotic cells were monitored with FACS after Annexin V and PI staining (*** *P*<0.001).

To understand the biological function of miR-195 in TSCC, miR-195 was overexpressed in TSCC cell lines. In the first of these experiments, we transfected TSCC cells with miR-195 and performed cell counting assays to evaluate the effects of miR-195 expression on cell proliferation and viability. Overexpression of miR-195 inhibited the viability of SCC-15 and CAL27 cells (Fig. 4A), leading to substantial accumulation of the cell population at the G1 stage of the cell cycle (Fig. 4B). Moreover, overexpression of miR-195 also promoted apoptosis in both cell lines (Fig. 4C).

### Overexpression of miR-195 Decreased Expression of Cyclin D1 and Bcl-2 by Targeting the 3′-UTRs of their mRNAs

**Figure 5:**
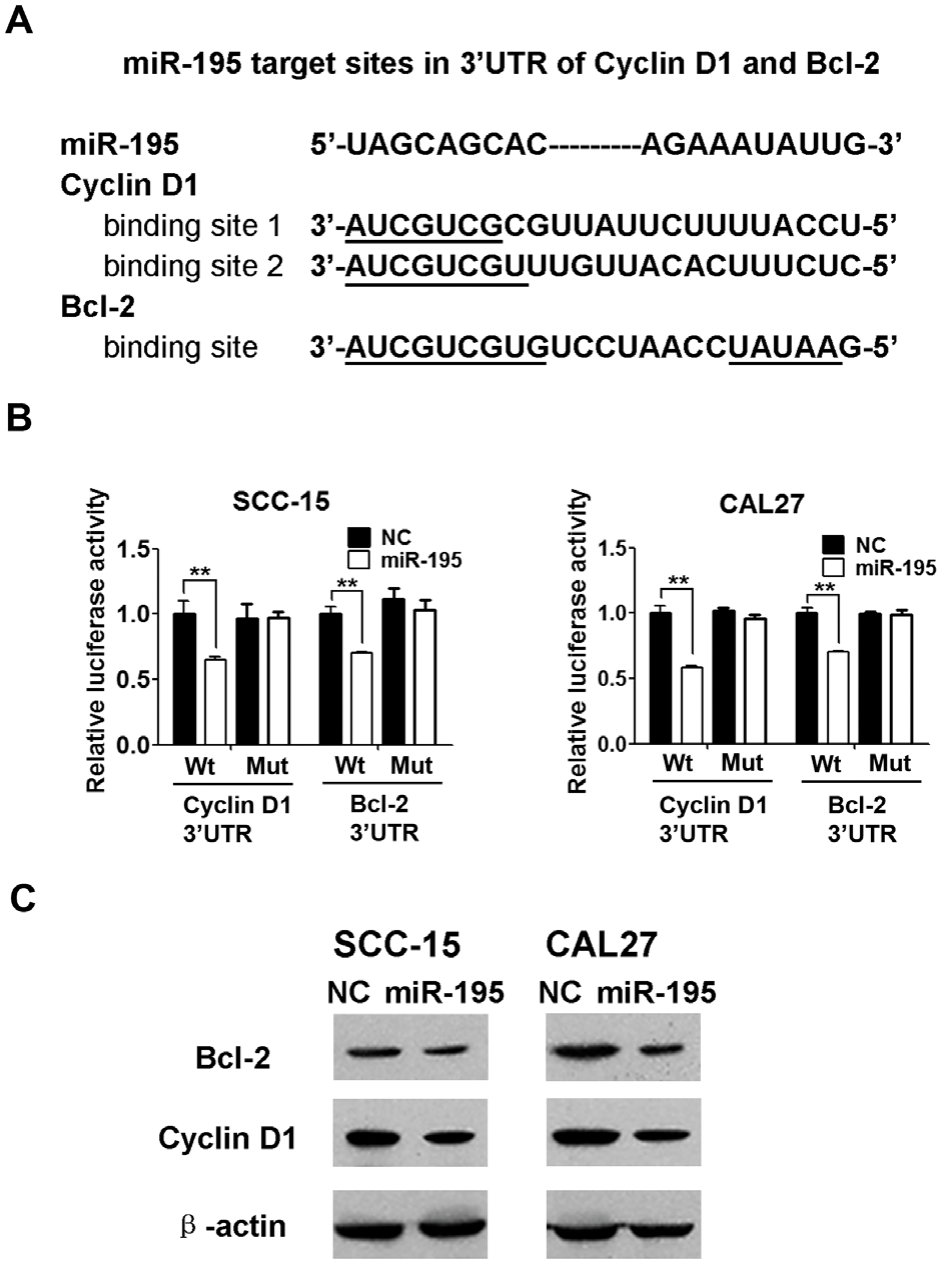
Cyclin D1 and Bcl-2 are direct targets of miR-195. (A), Sequence alignments of miR-195 and its target sites in 3′-UTRs of Cyclin D1 or Bcl-2. (B), Targeting of 3′-UTRs of Cyclin D1 or Bcl-2 by miR-195. SCC-15 and CAL27 cells were co-transfected with firefly luciferase reporter plasmids containing wildtype (Wt) and mutant wildtype and mutant (Mut) 3′-UTRs of Cyclin D1 or Bcl-2, and pRL-TK plasmid (a plasmid expressing rellina luciferase) and pcDNA3.0-miR-195 (miR-195) or pcDNA3.0 as indicated. After 48 h, firefly luciferase activities were measured and normalized by renilla luciferase activities. Data were presented as mean ± SD (n = 3) (**P<0.01). (C), Inhibition of protein expression of Cyclin D1 and Bcl-2. SCC-15 and CAL27 cells were transfected with pcDNA3.0 as a negative control (NC) or with pcDNA3.0-miR-195 (miR-195) as indicated. After 48 h, Cyclin D1, Bcl-2 and internal control β-actin were detected by Western blotting.

The mRNA for Cyclin D1 contains one conserved putative miR-195 target site in its 3′-UTR and that for Bcl-2 contains two conserved putative miR-195 target sites in its 3′-UTR, according to TargetScan predictions [16], [17] (Fig. 5A). Therefore, we constructed luciferase reporter plasmids to contain either the Cyclin D1 or the Bcl-2 3′-UTR sequence, including the wildtype and mutant miR-195 target sites. Firefly luciferase reporter containing wildtype or mutant 3′UTR of the target gene was cotransfected with renilla luciferase reporter and either pcDNA3.0 or pcDNA3.0-miR-195. Coexpression of pc3-miR-195 significantly suppressed firefly luciferase activity of the reporter with wildtype 3′UTR but not that of the mutant reporter (Fig. 5B). In addition, we examined the effects of overexpression of miR-195 on the endogenous expression of Cyclin D1 and Bcl-2 proteins in the two cell lines. Cyclin D1 and Bcl-2 expression were significantly decreased in SCC-15 and CAL27 cells in which miR-195 was overexpressed, in comparison with similar cells transfected with pcDNA3.0, a negative control (Fig. 5C). These findings further demonstrated that Cyclin D1 and Bcl-2 are direct targets of miR-195 in TSCC cell lines.

### Knockdown of the Endogenous Cyclin D1 or Bcl-2 Inhibited Cell Cycle Progression or Promoted Apoptosis in TSCC Cell Lines

**Figure 6:**
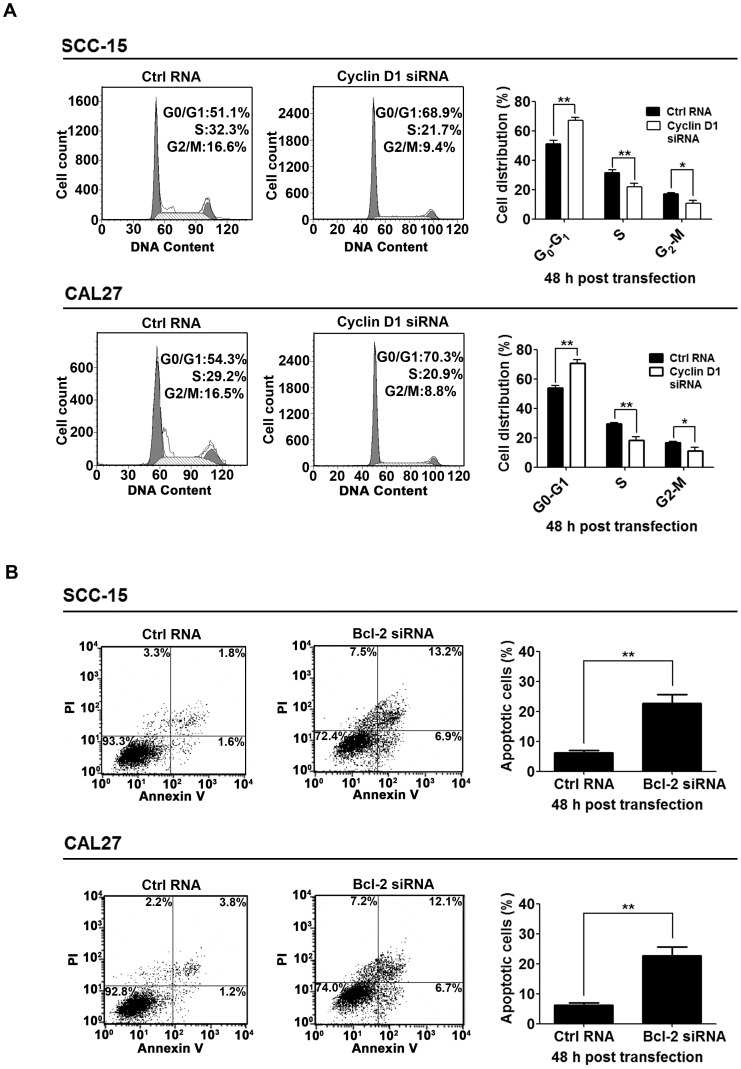
Inhibition of Cyclin D1 and Bcl-2 was responsible for the tumor suppressive effects of miR-195. (A), Inhibition of cell cycle progression by knockdown of Cyclin D1. SCC-15 and CAL27 cells were transfected with control RNA (Ctrl RNA) or Cyclin D1 siRNA as indicated. Cells were stained with propidium iodide (PI) at 48 h post-transfection and analyzed with FACS (* *P*<0.05, ** *P*<0.01). (B), Promotion of apoptosis by knockdown of Bcl-2. SCC-15 and CAL27 cells were transfected with control RNA (Ctrl RNA) or Bcl-2 siRNA as indicated. Apoptotic cells were monitored with FACS after Annexin V and PI staining (** *P*<0.01).

To ascertain the roles of Cyclin D1 and Bcl-2 in miR-195 regulated cell cycle progression and apoptosis, we determined if knockdown of the endogenous Cyclin D1 or Bcl-2 was able to mimic the effect of miR-195 restoration. We confirmed that Cyclin D1 knockdown inhibited cell cycle progression in TSCC cell lines, possibly be G1-phase cell cycle arrest (Fig. 6A). Knockdown of Bcl-2 also promoted apoptosis in TSCC cell lines (Fig. 6B). These data suggest that the antitumor effects of miR-195 may be mediated by inhibition of its target genes, Cyclin D1 and Bcl-2.

## Discussion

In this study, we observed that miR-195 expression was reduced in TSCC compared with adjacent nonmalignant tissues, and that decreased expression was correlated with cancer progression and prognosis. Moreover, we determined that decreased miR-195 expression was associated with poor overall survival in TSCC patients, independent of other clinicopathologic factors.

miR-195 could be a potential biomarker for prognosis prediction in TSCC patients. Except for their close association with patient outcomes, biomarkers should ideally be expressed at stable levels in the patient samples in question. In this respect, miRNAs are relatively stable as compared with other biological macromolecules. They can be well preserved in tissue samples even after formalin fixation and paraffin-embedding, and can be efficiently extracted and evaluated [28], [29]. Therefore, based on our observations that decreased miR-195 expression was associated with poor overall survival in TSCC patients, we anticipate that miR-195 could be a useful prognostic factor for TSCC and that its stability should allow the development of practical, economical methods for TSCC detection. Moreover, the development of cancers involves the altered expression of multiple genes, so the protein product of a single oncogene may not accurately reflect the status of the disease. However, just as other single miRNAs are known to target multiple messenger RNAs to regulate gene expression [30], miR-195 can also regulate multiple coding genes that are related to tumor growth [31]. Thus, expression of miR-195 is likely to reflect altered physiology of TSCC more precisely and effectively than any of the target genes alone.

Although our data failed to establish evidence for Cyclin D1 and Bcl-2 expression as prognostic markers in TSCC, we did demonstrate for the first time that immunostaining of Cyclin D1 and Bcl-2 is inversely correlated with miR-195 levels in TSCC tissues. Because Cyclin D1 and Bcl-2 have been shown to be direct targets of miR-195 [16], [17] and their expression in TSCC may account for the effect of miR-195, we examined the expression of these two proteins in paraffin sections of TSCC samples using immunohistochemistry. In this study, the expression of the Cyclin D1 was only statistically significantly associated with the tumor size of TSCC but not with other clinicopathological factors analyzed, whereas the expression of Bcl-2 in TSCC was not statistically significantly associated with any of the clinicopathological factors analyzed. However, according to previous studies, the role of Cyclin D1 as a prognostic marker remains controversial [32], [33], [34], [35], [36], [37] and there is no consensus on the use of Bcl-2 as a prognostic marker [38], [39], [40], [41] for squamous cell carcinomas among head and neck cancer patients either. Our results concerning Cyclin D1 and Bcl-2 were consistent with some of these publications [35], [36], [37], [38], [39]. Variation in the prognostic significance of Cyclin D1 and Bcl-2 in previous studies may be attributable to differences in sample size, definitions of positive expression, the inclusion of tumors from different subsites of the oral cavity, and the diversity of treatments. More importantly, our data showed that the expression of Cyclin D1 and Bcl-2 in TSCC tissues is inversely correlated with miR-195 levels. These important observations not only support previous findings that Cyclin D1 and Bcl-2 are target genes silenced by miR-195 but also demonstrate that the expression of miR-195 is potentially a more accurate prognostic tumor marker than Cyclin D1 or Bcl-2 levels alone in TSCC patients.

The anti-tumor effect of miR-195 in TSCC could be at least partially via inhibition of Cyclin D1 and Bcl-2 expression. We performed a series of experiments using two TSCC cell lines (SCC-15 and CAL27) to investigate the function of miR-195. Our results demonstrate that ectopic overexpression of miR-195 reduces cell viability, inhibits cell cycle progression, and promotes cell apoptosis. Moreover, Cyclin D1 and Bcl-2 were shown to be direct targets of miR-195 by a dual-luciferase reporter assay and western blots, and their inhibition may account for the antitumor effect of miR-195 in TSCC. However, because TargetScan predicts hundreds of potential targets of miR-195 (http://www.targetscan.org), we cannot exclude the possibility that other potential targets of miR-195 may govern additional cancer pathways that promote TSCC cancer development and that miR-195 may also target different molecules in different types of cancer.

Our study focused on a large series of patients who satisfied stringent recruitment criteria: (1) tumor location at the anterior body of the tongue, (2) squamous cell carcinoma, and (3) surgery as the primary treatment. We hope that this study will provide more accurate and clinically useful information on the prognostic significance of miR-195 expression. Several papers have described the involvement of miRNAs in head and neck squamous cell carcinoma [42], [43], [44], [45]. In these publications, which generally have included comparisons of normal and tumor samples, miRNA profiling was used to associate the expression of miRNAs with malignant progression and prognosis. Although these initial data have already suggested that miRNAs are involved in squamous cell carcinogenesis, the studies have always included heterogenous groups of patients with cancers from different subsites of oral cavity, and gene expression patterns from squamous cell carcinomas at different subsites of oral cavity may not be equally associated with cancer prognosis. For example, squamous cell carcinomas of the tongue have been shown to be different from those of the cheek in previous studies [46], [47], perhaps because different molecular genetic pathways are involved.

In conclusion, our study has confirmed in a large and homogeneous patient population that miR-195 expression was decreased in 80.2% of TSCC tumor samples compared with adjacent nonmalignant tissues and has provided evidence that miR-195 may be an independent biomarker of clinical prognosis among TSCC patients. Moreover, the anti-tumor effects of miR-195 in TSCC may be partially mediated by its inhibition of Cyclin D1 and Bcl-2 expression. Because miR-195 appears to have an anti-tumor effect in TSCC cell lines and has potential as a prognostic biomarker, it will be interesting in future experiments to further define the role of miR-195 in TSCC development.
